# Application of enhanced recovery after surgery during the perioperative period in children with Meckel’s diverticulum–a single-center prospective clinical trial

**DOI:** 10.3389/fped.2024.1378786

**Published:** 2024-03-19

**Authors:** Cuicui Wang, Youliang Wang, Ping Zhao, Ting Li, Fan Li, Zhi Li, Yingwen Qi, Xuewu Wang, Weidong Shi, Lina Liu, Gamei Li, Yong Wang

**Affiliations:** ^1^ Department of Pediatric Surgery, Gansu Provincial Maternal and Child Health Care Hospital, Lanzhou, China; ^2^ Department of Pediatric General Surgery, Gansu Provincial Maternal and Child Health Care Hospital, Lanzhou, China; ^3^ Department of Pediatric Urology Surgery, Gansu Provincial Maternal and Child Health Care Hospital, Lanzhou, China; ^4^ Department of Obstetrics, Gansu Provincial Maternal and Child Health Care Hospital, Lanzhou, China; ^5^ Department of Surgery, Gansu Provincial Maternal and Child Health Care Hospital, Lanzhou, China

**Keywords:** enhanced recovery after surgery, Meckel's diverticulum, pediatric, surgery, multimodal analgesia

## Abstract

**Background:**

Enhanced recovery after surgery (ERAS) has been widely used in adult surgery. However, few studies have reported the efficacy of ERAS in paediatric patients with Meckel's diverticulum (MD), the aim of the study was to prospectively evaluate the safety and efficacy of ERAS in treating MD.

**Methods:**

A prospective randomised controlled study of children with MD admitted to our hospital from Jan 1, 2021 to Dec 31, 2023 were conducted, we developed and implemented an ERAS program for this patients. All cases were strictly selected according to the inclusion and exclusion criteria. Among these patients, they were randomly assigned to the ERAS group or the traditional (TRAD) group with random number table row randomization. The main observational indicators were operation time, intraoperative hemorrhage, FLACC pain scale results on 2 h, 6 h, 12 h, 24 h after surgery, length of postoperative stay (LOPS), time to first defecation, time to first eating after surgery, time to discontinuation of intravenous infusion, total treatment cost, incidence of postoperative complications, 30-day readmission rate and parental satisfaction rate.

**Results:**

A total of 50 patients underwent Meckel's diverticulectomy during this period, 7 patients were excluded, 23 patients were assigned to the ERAS group and 20 patients were assigned to the TRAD group. There were no significant differences in demographic data and operation time, intraoperative hemorrhage. The FLACC pain scale results on 2 h, 6 h, 12 h, 24 h after surgery were significantly lower in the ERAS group. The LOPS was 6.17 ± 0.89 days in the ERAS group and 8.30 ± 1.26 days in the TRAD group, resulting in a significantly shorter LOPS in ERAS group. ERAS could also reduce the first postoperative defecation time, the time to first eating after surgery and the time to discontinuation of intravenous infusion. The treatment cost was decreased in the ERAS group. The rate of complications and 30-day readmission were not significantly different between the two groups.

**Conclusions:**

In this single-center study, the ERAS protocol for patients with MD requiring surgery was safe and effective.

## 1 Introduction

ERAS is a series of perioperative optimisation measures under multidisciplinary collaboration aimed at alleviate patients’ pain, reduce perioperative psychological and physiological stress, and reduce the occurrence of postoperative complications, thereby accelerating patients’ postoperative recovery (1). ERAS was first used in adult colorectal surgery and is now widely accepted by adult surgeons. ERAS has been progressively used in paediatric surgery recent years, but there are not many high-quality literature on the implementation of ERAS programs (2). Children experience a more complex surgical stress response than adults. Therefore, optimising perioperative management in children is particularly important and urgent.

Meckel's diverticulum (MD) is caused by incomplete obliteration of the viteline duct during embryonic (3), it is a common congenital intestinal malformation accounting for half of pediatric gastrointestinal bleeds in childhood, with a prevalence in the general population of approximately 2% to 4% (4). In most cases it is clinically silent and found incidentally during abdominal exploration for other pathologies. Most patients are diagnosed due to complications such as gastrointestinal bleeding, intussusception, intestinal obstruction or diverticulitis, which require surgical treatment once detected (5, 6). With the popularization of minimally invasive surgical techniques, laparoscopic-assisted Meckel's diverticulectomy has become a popular surgical procedure (7, 8).

However, few studies have reported the efficacy of ERAS in paediatric MD patients. We conducted a prospective single-center randomized study to investigate the safety and efficacy of ERAS on the treatment of MD.

## 2 Materials and methods

### 2.1 Study design

#### 2.1.1 General information

The study was a prospective, single-center randomized controlled study of an ERAS program adapted for children with MD undergoing operations from Jan 1, 2021 to Dec 31, 2023. All cases were strictly selected according to the inclusion and exclusion criteria. Among these patients, they were randomly assigned to the ERAS group or the traditional (TRAD) group with random number table row randomization. The TRAD group was the control group. This study was approved by the Ethics Committee of Gansu Maternal and Child Health Hospital (No. (2022)GSFY Review (29)), and informed consent was obtained from family members.

Inclusion criteria:

1. Previous history of blood in stool, and obtained a clear diagnosis by isotopic ectopic gastric mucosal imaging, B-mode ultrasound or small bowel microscopy;
2. Isotopic ectopic gastric mucosal imaging, B-mode ultrasound or small bowel microscopy did not obtain a clear diagnosis, after the conservative treatment by the internal medicine, the blood in stool is still recurrent, and highly suspected of MD;
3. Postoperative pathological diagnosis of MD.

Exclusion criteria:

1. Age >7 years, or <3 years;
2. Previous history of abdominal surgery;
3. Patients with MD in combination with other abdominal surgeries;
4. Comorbidity with other serious diseases that may affect rehabilitation;
5. Serious postoperative complications that make continuation of the ERAS programme unsuitable.

#### 2.1.2 Surgical methods

Both groups underwent laparoscopic-assisted transumbilical Meckel's diverticulectomy as reported in the literature (9). All procedures were performed by the same surgical and anesthesia team, and the surgeon had more than 3 years of experience in laparoscopic surgery.

#### 2.1.3 Observation indicators

The main observational indicators were operation time, intraoperative hemorrhage, FLACC pain scale results on 2 h, 6 h, 12 h, 24 h after surgery, length of postoperative stay (LOPS), time to first defecation, time to first eating after surgery, time to discontinuation of intravenous infusion, total treatment cost, incidence of postoperative complications, 30-day readmission rate and parental satisfaction rate.

Parental satisfaction rate was captured from parents when the children were discharged from hospital. We takes a 5-level grading methods for quantification, which is divided into “very satisfied, satisfied, average, dissatisfied and very dissatisfied”. If the parents choose “satisfied” or “very satisfied”, we consider that they were satisfied with the treatment during hospitalization.

### 2.2 Perioperative management

The development of the ERAS program includes elements of pre-operative, intraoperative and post-operative care. The main differences between the ERAS and TRAD programs are shown in Table 1. Additionally, in the ERAS group, 2 ml/kg of 5% GS was given orally every 3 h after 2 h of awakening from anaesthesia, and a residue-free diet was given on the first postoperative day, followed by a liquid diet on the second day, and gradually transitioning to a normal diet. In the TRAD group, patients were fasted for 3–5 days after surgery, starting with 5% GS and then gradually transitioning to a normal diet. If patient could tolerate oral feeding, the infusion was discontinued. For anaesthesia, dexmedetomidine continuous intravenous pumping combined with 0.25% ropivacaine injection 0.5 mg/kg local incision infiltration was used in the ERAS group. The FLACC pain rating scale was used for pain assessment at 2 h, 6 h, 12 h, 24 h postoperatively, respectively. Ibuprofen suppositories were used for analgesia when the FLACC pain score was ≥7.

**Table 1:** Difference in perioperative care between the ERAS and TRAD groups.

		TRAD group	ERAS group
Pre-operative	Perioperative counseling	Non-compulsory requirement	Through pictures, videos and other means of education (by surgeons, anesthesiologists, and surgical ward nurses)
	Preoperative fasting	No food or drink for 8 h before surgery	Fasting from milk or formula for 6 h, breast milk for 4 h; Drinking 10% glucose solution or water(10 ml/kg, 2 h prior to anesthesia)
Intraoperative	Preoperative antibiotics	Within 60 min prior to incision	Within 60 min prior to incision
	Anesthesia	General anesthesia	General anesthesia + dexmedetomidine continuous intravenous pumping during anesthesia combined with 0.25% ropivacaine injection 0.5 mg/kg local incision infiltration
	Fluid management	No	Yes (Goal-oriented, controlled infusion based on heart rhythm, blood pressure, and urine output)
	Temperature anagement	Control operating room temperature	Control operating room temperature; Heating of operating beds with heating blankets; Heating of intraoperative flushes and intravenous fluids
	Abdominal drainage tube	Used, removed on POD 2–3	Not used unless they had much exudation during surgery
Post-operative	Nasogastric tube	Used, removed on POD 2–3	Not used unless delayed gastric emptying occurs
	Urethral tube	Used, removed on POD 1–2	Not used
	Postoperative fasting	No, oral intake for 3–5 days	Early feeding
	Get out of bed early	No (Postoperative bed rest for 1–2 days)	Mobilization (on POD 1)
	Early removal of intravenous fluids	No	Yes (Gradually reduce intravenous fluid according to the amount of food intake, the infusion was stopped on POD 5–6)

Discharge criteria: fully tolerated orally; intravenous fluids stopped; good healing of surgical incisions; normal urination and defecation; no fever; no abdominal pain and none of the other complications.

### 2.3 Follow-up

After discharge, the nurse will follow up by telephone. If there was any discomfort such as fever or abdominal pain, the patient would be suggested to come back for examination. The follow-up time was 6 months.

### 2.4 Statistics

SPSS version 26.00 was applied for statistical analysis. Normally distributed measurements were described by median and standard deviations, and independent samples t-test was used for comparison; Non-normally distributed data were described by median and quartiles, and Mann-Whitney u-test was used for comparison. Count data were compared using the *χ*^2^ test or Fisher's exact probability method. All tests were two-tailed and *P* < 0.05 was considered a statistically significant difference.

## 3 Results

**Figure 1:**
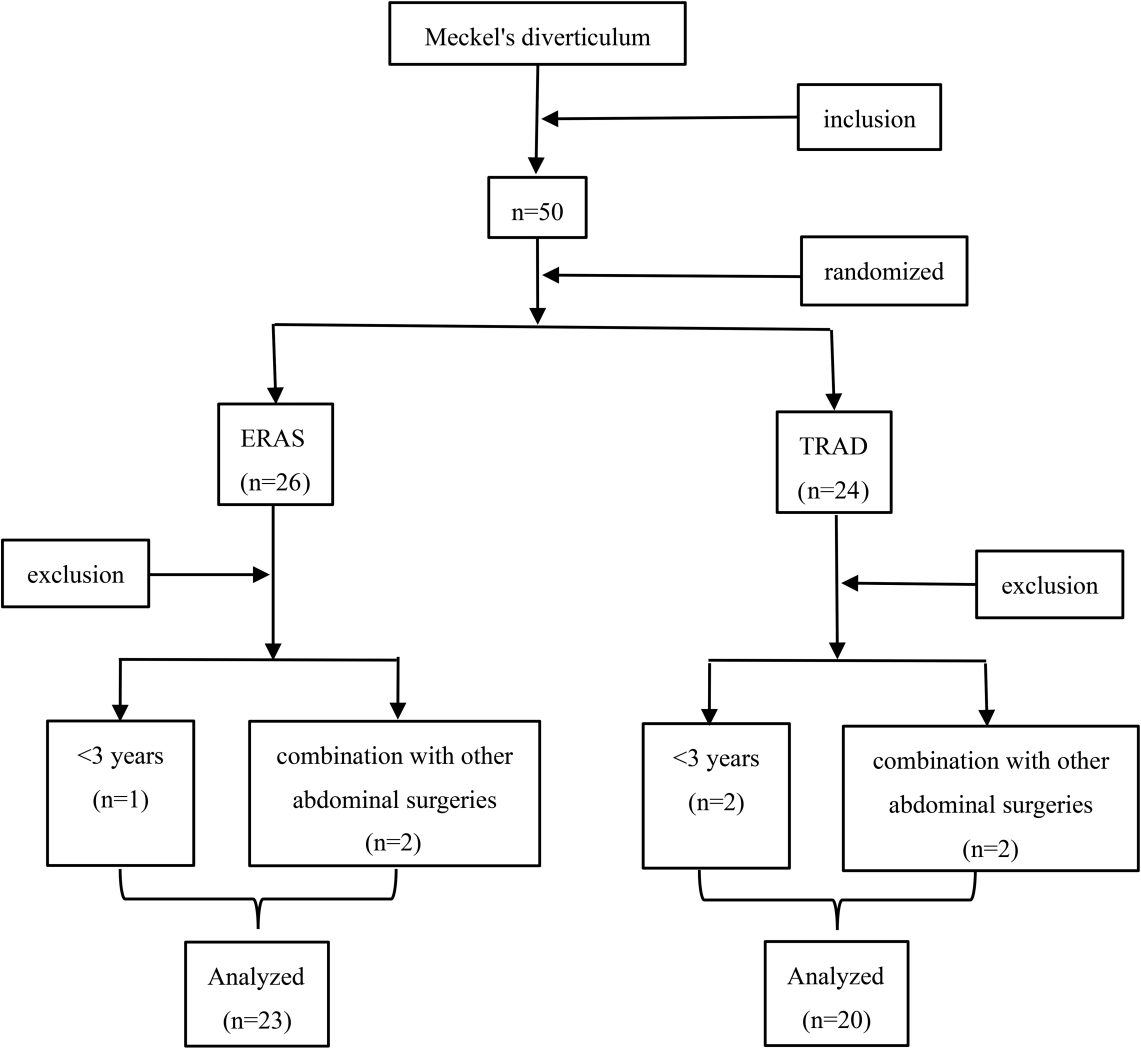
Screening flow chart of this study.

A total of 50 patients underwent Meckel's diverticulectomy during this period, 7 patients were excluded, 23 patients were assigned to the ERAS group and 20 patients were assigned to the TRAD group (Figure 1). The general information of the patients is shown in Table 2. There was no statistically significant comparison of age, weight and gender between the two groups (*P *> 0.05) (Table 2).

**Table 2:** General information of the patients.

	TRAD group	ERAS group	*P* value
Number of patients	20	23	
Age (years)	4.50 ± 1.28	4.74 ± 1.51	0.58
Weight (kg)	19.05 ± 3.32	19.43 ± 3.01	0.69
Sex			>0.05
Males	18	19	
Females	2	4	

There were no statistically significant difference in operation time and intraoperative hemorrhage between the two groups. The FLACC pain scale results on 2 h, 6 h, 12 h, 24 h after surgery were significantly lower in the ERAS group than in the TRAD group (*P *< 0.001) (Table 3).

**Table 3:** Outcomes in the ERAS group compared to the TRAD group.

	TRAD group	ERAS group	*P* value
Intraoperative operation time (min)	63.50 ± 10.27	63.91 ± 11.67	0.90
Intraoperative hemorrhage(ml)	4.90 ± 1.99	4.83 ± 1.40	0.89
FLACC pain scale results on 2 h after surgery	3.05 ± 0.61	1.43 ± 0.84	<0.001
FLACC pain scale results on 6 h after surgery	4.35 ± 0.75	2.22 ± 0.60	<0.001
FLACC pain scale results on 12 h after surgery	6.60 ± 1.05	2.78 ± 0.95	<0.001
FLACC pain scale results on 24 h after surgery	4.45 ± 0.61	2.09 ± 0.85	<0.001
Time to first defecation (h)	40.70 ± 7.20	20.65 ± 4.07	<0.001
Time to first eating after surgery (days)	3.85 ± 0.75	1.61 ± 0.50	<0.001
Time to discontinuation of intravenous infusion (days)	6.95 ± 1.32	5.39 ± 0.84	<0.001
Length of postoperative stay (days)	8.30 ± 1.26	6.17 ± 0.89	<0.001
Treatment cost(yuan)	22,672.05 ± 2,397.80	17,673.01 ± 2,156.61	<0.001
Postoperative complications, *n* (%)	2/20	1/23	>0.05
30-day readmissions, *n* (%)	0	0	>0.05
Parental satisfaction rate (%)	90%	96%	>0.05

The LOPS was 6.17 ± 0.89 days in the ERAS group and 8.30 ± 1.26 days in the TRAD group, resulting in a significantly shorter LOPS in the ERAS group than in the control group (*P *< 0.001) (Table 3).

ERAS could also reduce the first postoperative defecation time (*P* < 0.001), the time to first eating after surgery (*P *< 0.001) and the time to discontinuation of intravenous infusion (*P *< 0.001). The treatment cost was decreased in the ERAS group (*P *< 0.001). The rate of complications (*P *> 0.05) and 30-day readmission (*P *> 0.05) were not significantly different between the two groups. ERAS group has a higher parental satisfaction rate, although there was no statistical difference (96% vs. 90%). There were no readmissions or deaths in the two groups (Table 3).

## 4 Discussion

In the late 1990s, Danish surgeon Kehlet first proposed the concept of ERAS (10). ERAS program is mainly achieved through preoperative, intraoperative, and postoperative measures (11). Preoperative measures mainly include perioperative counseling, minimized preoperative fasting; Intraoperative measures mainly include optimal anesthesia, minimally invasive techniques, temperature management, infusion management; Postoperative measures mainly include minimized postoperative fasting, early postoperative mobilization, limited intravenous fluids, and early removal of surgical tubes (12, 13). The results of this study showed that compared with the traditional perioperative management, ERAS was used to significantly reduce pain in children with laparoscopic-assisted Meckel's diverticulectomy. The ERAS group had an advantage over the TRAD group in terms of LOPS, the time of first defecation after operation, the time to first eating after surgery, the time to discontinuation of intravenous infusion, the treatment cost, and the incidence of postoperative complications and 30-day readmission rate did not increase, which suggests that ERAS can be safely and effectively applied to children with MD.

### 4.1 Preoperative management

The child’s anxiety and fear usually stems from unfamiliarity with the environment, whereas the parents’ anxiety often stems from a lack of knowledge about the disease and fear of surgery (14). Perioperative counseling provided by surgeons, anesthesiologists, and surgical ward nurses is very important, can provide relevant knowledge about MD, may alleviate children's and their parents’ psychological stress, and motivate participate in the perioperative care such as early mobilization and oral feeding. In our study, families were actively cooperating with the ERAS program. Parental satisfaction rate in the EARS group was 96%, which was higher than 90% in the TRAD group, although there was no statistical difference.

The changes of the fasting concept was the earliest measure proposed by ERAS. Long preoperative fasting can lead to dehydration, hypoglycemia, and anxiety in children (15, 16). According to the recommendations by the American society of anesthesiologists in 2017, clear liquids may be ingested for up to 2 h, breast milk for up to 4 h, infant formula and light meals or non-human milk may be ingested for up to 6 h before procedures requiring general anesthesia, regional anesthesia, or procedural sedation and analgesia (17). In this study, the ERAS group shortened the preoperative fasting time, relieved hunger, improved the comfort of patients, and alleviated anxiety among the children's families.

### 4.2 Intraoperative management

Pain is considered as the fifth vital sign (18).The pain regulation system of children is not as well developed as that of adults, and the level of stress response after surgery is approximately 3–5 times higher than that of adults (19). The wound pain after abdominal surgery restricts the respiratory movement, making it difficult to cough and expel airway secretions, pulmonary infections are prone to occur. At the same time, pain makes children cry and become irritable, delayis early postoperative mobilization time and affects wound healing. Single analgesic measure cannot achieve good analgesic effects, it is necessary to combine different analgesic drugs or different analgesic methods to make up for the inadequacy of a single analgesic drug or analgesic method (20, 21). The Guidelines for Perioperative Care in Elective Colorectal Surgery (2018) clearly states that postoperative analgesia should adopt a Multimodal Analgesia (MMA) Management (12).

MMA specifically recommends local analgesic techniques and believes that invasive local anesthesia through incisions is necessary for either open or laparoscopic surgery (22). Dexmedetomidine is a highly selective α2-adrenoreceptor agonist with sedative, analgesic, anxiolytic and sympatholytic without causing respiratory depression, it is commonly used in MMA (23). In this study, a multimodal analgesic regimen of local anesthesia combined with dexmedetomidine was used in the ERAS group, we found that the scores at 2 h, 6 h, 12 h, and 24 h after surgery in the ERAS group were lower than those in the TRAD group, indicating that the MMA patterns effectively alleviate postoperative pain in children and promote early mobilization after surgery.

Studies have shown that low temperature in the perioperative period can cause abnormal coagulation function, which can easily lead to the occurrence of complications such as surgical site infection, delayed wound healing, and cardiac arrhythmia (24), and the use of warm mattresses and heaters to maintain the child's core body temperature of no less than 36 °C can reduce intraoperative hemorrhage, shorten the operation time, and accelerate the recovery after the operation.

### 4.3 Postoperative management

Research has found that minimally invasive laparoscopic surgery has lesser interference on the intestines than traditional open surgery, and the gastrointestinal function can be returned to normal in 1–2 days postoperatively. Nasogastric tube is not routinely used unless delayed gastric emptying occurs (12, 25). In addition, prolonged indwelling urinary tube not only increase the discomfort of children, but also increase the incidence of urinary tract infections. Postoperative prophylactic abdominal drainage in patients undergoing elective abdominal surgery does not reduce the incidence or severity of anastomotic leaks and other complications, therefore, centres with mature laparoscopic techniques may consider omitting abdominal drainage (26). In our study, nasogastric and urinary tubes were not routinely used in the ERAS group, nor did they place abdominal drainage tubes unless they had much exudation during surgery. No discomfort such as vomiting, abdominal distention, urinary retention, and no complications such as anastomotic leakage occurred after operation.

Almost all ERAS studies have measures related to early postoperative feeding. Regarding the choice of feeding time after intestinal surgery, most surgeons still follow the traditional principle of fasting for one week after surgery and then gradually starting eating. A study has found that fasting for more than 24 h after surgery leads to weakened muscle function and significantly increased insulin resistance in the body (27). In addition, prolonged fasting may impair immune function, affecting postoperative recovery and wound healing in children (28). Recent systematic review suggested that early enteral feeding within 24 h after abdominal surgery is safe and effective in children, can maintain intestinal nutrient absorption, also reduce the risk of any type of infection and the mean LOPS (29, 30). Studies have also shown that exercise can relieve pain and accelerate recovery of bowel function (31). Therefore, ERAS advocates starting enteral nutrition and postoperative mobilization as early as possible (32, 33). In our study, children in ERAS group were allowed to drink water on the first day after awakening from anaesthesia, and then gradually transitioned to a normal diet, and started getting out of bed on the first postoperative day. The time to first defecation and the time to first eating after surgery was advanced in the EARS group. It also shortened the postoperative intravenous infusion time and hospitalization time, reduced total treatment cost, and the incidence of postoperative complications did not increase.

Recovery of gastrointestinal function is an independent predictor of postoperative recovery. Excessive intraoperative fluid infusion not only leads to tissue oedema and increases cardiovascular and respiratory burdens, but also significantly affects the recovery of gastrointestinal function (34). The fundamental aim of fluid therapy is to maintain good tissue perfusion, both to avoid tissue hypoperfusion due to volume insufficiency and to avoid adverse effects from volume overload. During our surgery, the ERAS group used goal-directed fluid therapy and made appropriate adjustments based on urine output, intraoperative bleeding volume, and hemodynamic parameters (35). Once the patient can tolerate a normal diet after surgery, intravenous infusion were stopped in children. In this study, the time to first defecation was significantly shorter in the ERAS group, indicating that the ERAS programme resulted in a quicker recovery of gastrointestinal function.

However, this study has limitations such as single-centre design, small sample size and short follow-up time. Further expansion of the sample size and longer follow-up time are needed to investigate the applicability of this ERAS protocol to the paediatric population.

## 5 Conclusion

Thus, a combination of multiple perioperative interventions is more conducive to rapid postoperative recovery than separate interventions. In this single-centre study, the implementation of ERAS in MD surgery was feasible and safe, and shortened LOPS without increasing the incidence of complications.

## Data Availability

The original contributions presented in the study are included in the article/Supplementary Material, further inquiries can be directed to the corresponding author.

## References

[1] Rollins KE Lobo DN Joshi GP. Enhanced recovery after surgery: current status and future progress. Best Pract Res Clin Anaesthesiol. (2021) 35(4):479–89. 10.1016/j.bpa.2020.10.001 34801211

[2] Leeds IL Boss EF George JA Strockbine V Wick EC Jelin EB. Preparing enhanced recovery after surgery for implementation in pediatric populations. J Pediatr Surg. (2016) 51(12):2126–29. 10.1016/j.jpedsurg.2016.08.029 27663124 PMC5373552

[3] Stănescu GL Pleşea IE Diaconu R Gheonea C Sabetay C Ţîştea D Meckel's diverticulum in children, clinical and pathological aspects. Rom J Morphol Embryol. (2014) 55(3):1167–70. 25607401

[4] Sagar J Kumar V Shah DK. Meckel's diverticulum: a systematic review. J R Soc Med. (2006) 99(10):501–505. 10.1177/014107680609901011 17021300 PMC1592061

[5] Lindeman RJ Søreide K. The many faces of Meckel's diverticulum: update on management in incidental and symptomatic patients. Curr Gastroenterol Rep. (2020) 22(1):3. 10.1007/s11894-019-0742-1 31930430

[6] Itriyeva K Harris M Rocker J Gochman R. Not just painless bleeding: Meckel's diverticulum as a cause of small bowel obstruction in children-two cases and a review of the literature. Case Rep Emerg Med. (2015) 2015:938346. 10.1155/2015/938346 26788380 PMC4695658

[7] Skertich NJ Ingram MC Grunvald MW Williams MD Ritz E Shah AN Outcomes of laparoscopic versus open resection of Meckel's diverticulum. J Surg Res. (2021) 264:362–67. 10.1016/j.jss.2021.02.028 33848834

[8] Redman EP Mishra PR Stringer MD. Laparoscopic diverticulectomy or laparoscopic-assisted resection of symptomatic Meckel diverticulum in children? A systematic review. Pediatr Surg Int. (2020) 36(8):869–74. 10.1007/s00383-020-04673-5 32436063

[9] Papparella A Nino F Noviello C Marte A Parmeggiani P Martino A Laparoscopic approach to Meckel's diverticulum. World J Gastroenterol. (2014) 20(25):8173–78. 10.3748/wjg.v20.i25.8173 25009390 PMC4081689

[10] Kehlet H. Multimodal approach to control postoperative pathophysiology and rehabilitation. Br J Anaesth. (1997) 78(5):606–17. 10.1093/bja/78.5.606 9175983

[11] Do-Wyeld M Rogerson T Court-Kowalski S Cundy TP Khurana S. Fast-track surgery for acute appendicitis in children: a systematic review of protocol-based care. ANZ J Surg. (2019) 89(11):1379–85. 10.1111/ans.15125 30989778

[12] Gustafsson UO Scott MJ Hubner M Nygren J Demartines N Francis N Guidelines for perioperative care in elective colorectal surgery: enhanced recovery after surgery (ERAS®) society recommendations: 2018. World J Surg. (2019) 43(3):659–95. 10.1007/s00268-018-4844-y 30426190

[13] Cavallaro P Bordeianou L. Implementation of an ERAS pathway in colorectal surgery. Clin Colon Rectal Surg. (2019) 32(2):102–108. 10.1055/s-0038-1676474 30833858 PMC6395097

[14] Getahun AB Endalew NS Mersha AT Admass BA. Magnitude and factors associated with preoperative anxiety among pediatric patients: cross-sectional study. Pediatric Health Med Ther. (2020) 11:485–94. 10.2147/PHMT.S288077 33364873 PMC7751437

[15] Brady M Kinn S Ness V O'Rourke K Randhawa N Stuart P. Preoperative fasting for preventing perioperative complications in children. Cochrane Database Syst Rev. (2009) (4):CD005285. 10.1002/14651858.CD005285.pub2 19821343

[16] Cook-Sather SD Litman RS. Modern fasting guidelines in children. Best Pract Res Clin Anaesthesiol. (2006) 20(3):471–81. 10.1016/j.bpa.2006.02.003 17080697

[17] The American Society of Anesthesiologists. Practice guidelines for preoperative fasting and the use of pharmacologic agents to reduce the risk of pulmonary aspiration:application to healthy patients undergoing elective procedures:an updated report by the American society of anesthesiologists task force on preoperative fasting and the use of pharmacologic agents to reduce the risk of pulmonary aspiration. Anesthesiology. (2017) 126(3):376–93. 10.1097/ALN.0000000000001452 28045707

[18] Morone NE Weiner DK. Pain as the fifth vital sign: exposing the vital need for pain education. Clin Ther. (2013) 35(11):1728–32. 10.1016/j.clinthera.2013.10.001 24145043 PMC3888154

[19] McPherson C Grunau RE. Neonatal pain control and neurologic effects of anesthetics and sedatives in preterm infants. Clin Perinatol. (2014) 41(1):209–27. 10.1016/j.clp.2013.10.002 24524456 PMC3925313

[20] Chou R Gordon DB de Leon-Casasola OA Rosenberg JM Bickler S Brennan T Management of postoperative pain: a clinical practice guideline from the American pain society, the American society of regional anesthesia and pain medicine, and the American society of Anesthesiologists’ committee on regional anesthesia, executive committee, and administrative council. J Pain. (2016) 17(2):131–57. 10.1016/j.jpain.2015.12.008 26827847

[21] Wick EC Grant MC Wu CL. Postoperative multimodal analgesia pain management with nonopioid analgesics and techniques: a review. JAMA Surg. (2017) 152(7):691–97. 10.1001/jamasurg.2017.0898 28564673

[22] Beverly A Kaye AD Ljungqvist O Urman RD. Essential elements of multimodal analgesia in enhanced recovery after surgery (ERAS) guidelines. Anesthesiol Clin. (2017) 35(2):e115–43. 10.1016/j.anclin.2017.01.018 28526156

[23] Ustun YB Turunc E Ozbalci GS Dost B Bilgin S Koksal E Comparison of ketamine, dexmedetomidine and lidocaine in multimodal analgesia management following sleeve gastrectomy surgery: a randomized double-blind trial. J Perianesth Nurs. (2022) 37(6):820–26. 10.1016/j.jopan.2021.12.012 35382963

[24] Nemeth M Miller C Bräuer A. Perioperative hypothermia in children. Int J Environ Res Public Health. (2021) 18(14):7541. 10.3390/ijerph18147541 34299991 PMC8308095

[25] Davila-Perez R Bracho-Blanchet E Tovilla-Mercado JM Hernandez-Plata JA Reyes-Lopez A Nieto-Zermeño J. Unnecessary gastric decompression in distal elective bowel anastomoses in children: a randomized study. World J Surg. (2010) 34(5):947–53. 10.1007/s00268-010-0442-3 20140434

[26] Ma MKI Chung PHY Yeung F Wong KKY. Analysing factors prolonging hospital stay after excision of choledochal cyst-A pathway towards enhanced recovery after surgery. World J Surg. (2023) 47(12):3012–19. 10.1007/s00268-023-07206-y 37816975

[27] Herbert G Perry R Andersen HK Atkinson C Penfold C Lewis SJ Early enteral nutrition within 24 hours of lower gastrointestinal surgery versus later commencement for length of hospital stay and postoperative complications. Cochrane Database Syst Rev. (2019) 7(7):CD004080. 10.1002/14651858.CD004080.pub4 31329285 PMC6645186

[28] Weimann A Braga M Carli F Higashiguchi T Hübner M Klek S ESPEN practical guideline: clinical nutrition in surgery. Clin Nutr. (2021) 40(7):4745–61. 10.1016/j.clnu.2021.03.031 34242915

[29] Greer D Karunaratne YG Karpelowsky J Adams S. Early enteral feeding after pediatric abdominal surgery: a systematic review of the literature. J Pediatr Surg. (2020) 55(7):1180–87. 10.1016/j.jpedsurg.2019.08.055 31676081

[30] Issac A Dhiraaj S Halemani K Thimmappa L Mishra P Kumar B Efficacy of early enteral nutrition on gastrointestinal surgery outcomes: a systematic review and meta-analysis. Eur J Pediatr Surg. (2023) 33(6):454–62. 10.1055/s-0043-1760837 36724826

[31] Pedersen L Idorn M Olofsson GH Lauenborg B Nookaew I Hansen RH Voluntary running suppresses tumor growth through epinephrine- and IL-6-dependent NK cell mobilization and redistribution. Cell Metab. (2016) 23(3):554–62. 10.1016/j.cmet.2016.01.011 26895752

[32] Srinivasa S Lemanu DP Singh PP Taylor MH Hill AG. Systematic review and meta-analysis of oesophageal Doppler-guided fluid management in colorectal surgery. Br J Surg. (2013) 100(13):1701–1708. 10.1002/bjs.9294 24227354

[33] Bevilacqua LA Obeid NR Spaniolas K Bates A Docimo S Jr Pryor A. Early postoperative diet after bariatric surgery: impact on length of stay and 30-day events. Surg Endosc. (2019) 33(8):2475–78. 10.1007/s00464-018-6533-1 30374793

[34] Malbrain MLNG Langer T Annane D Gattinoni L Elbers P Hahn RG Intravenous fluid therapy in the perioperative and critical care setting: executive summary of the International Fluid Academy (IFA). Ann Intensive Care. (2020) 10(1):64. 10.1186/s13613-020-00679-3 32449147 PMC7245999

[35] Sun Y Chai F Pan C Romeiser JL Gan TJ. Effect of perioperative goal-directed hemodynamic therapy on postoperative recovery following major abdominal surgery-a systematic review and meta-analysis of randomized controlled trials. Crit Care. (2017) 21(1):141. 10.1186/s13054-017-1728-8 28602158 PMC5467058

